# Maintaining agency during adolescent cancer: Retrospective narratives among young adult survivors — A qualitative content analysis

**DOI:** 10.1016/j.apjon.2026.100997

**Published:** 2026-06-18

**Authors:** Marzieh HelalBirjandi, Gholamhossein Mahmoudirad, Ali Ghasemi, Ahmad Nasiri

**Affiliations:** a Department of Nursing, Birjand University of Medical Sciences, Birjand, Iran; b Department of Pediatrics Hematology and Oncology, Mashhad University of Medical Sciences, Mashhad, Iran; c Birjand Health Qualitative Research Center, Birjand University of Medical Sciences, Birjand, Iran

**Keywords:** Adolescent cancer survivors, Qualitative content analysis, Coping, Social relationships, Personal autonomy

## Abstract

**Objective:**

A cancer diagnosis during adolescence can disrupt psychosocial development, autonomy, and everyday social life. This study retrospectively described young adult cancer survivors’ experiences of coping with cancer and efforts to maintain personal agency and active involvement during treatment.

**Methods:**

This qualitative study (2024–2025) was conducted in eastern Iran using conventional content analysis. Fifteen young adult cancer survivors were recruited through purposive and snowball sampling methods. Participants aged 18–29 years had been diagnosed with cancer between the ages of 11 and 18 years. All had completed treatment at least two years before participation. Data were collected through in-depth semi-structured interviews and analyzed using the approach described by Graneheim and Lundman.

**Results:**

Analysis generated four main categories and 14 subcategories: sustaining hope despite suffering, maintaining control under illness constraints, managing social relationships, and managing life responsibilities. Participants retrospectively described coping as an active and ongoing process to sustain hope, preserve a sense of agency, maintain social connections, and uphold continuity in everyday life despite treatment-related challenges.

**Conclusions:**

Young adult survivors portrayed their adolescent cancer experiences as involving active engagement with the psychological, social, and practical challenges of illness. These findings highlight the importance of recognizing adolescents as active participants in their cancer care and supporting their involvement, communication, and psychosocial needs throughout treatment.

## Introduction

Childhood and adolescent cancers, typically defined as cancers diagnosed between birth and 19 years of age, remain important global health concerns.^1^ Although they continue to impose a substantial health burden worldwide, including in Iran,^2^^,^^3^ advances in diagnosis and treatment have improved survival rates over recent decades.^4^ While population-based analyses using SEER data have reported five-year survival rates exceeding 80% have been reported for pediatric cancers in high-income settings / in SEER-based analyses,^4^ survival rates in low- and middle-income countries remain lower, reflecting persistent disparities in access to timely diagnosis and treatment.^5^ The World Health Organization’s Global Initiative for Childhood Cancer aims to achieve a global survival rate of at least 60% by 2030.^1^

These advances have resulted in a growing population of cancer survivors. The National Cancer Institute conceptualizes cancer survivorship as beginning at diagnosis and continuing throughout life.^6^ Mullan’s Seasons of Survival framework describes survivorship across acute, extended, and permanent phases.^7^ Although such frameworks help understand survivorship trajectories, they may not fully capture how individuals later interpret and reconstruct illness experiences within everyday life. This limitation becomes especially relevant when cancer occurs during adolescence.

Adolescence represents a critical developmental period characterized by identity formation, increasing autonomy, and the expansion of social relationships; accordingly, a cancer diagnosis during this stage can significantly disrupt normative psychosocial development.^8^ Evidence suggests that such a diagnosis may be accompanied by feelings of uncertainty, fear, loneliness, and social isolation.^9^^,^^10^ Studies conducted in Iran have indicated that cancer-related stigma and negative social perceptions may lead to illness concealment and difficulties in interpersonal communication among individuals with cancer.^11^ These developmental and sociocultural factors underscore the importance of understanding how cancer disrupts adolescent life and how adolescents actively respond to illness-related challenges.

Previous research suggests that adolescents use a variety of coping strategies to manage the psychological and emotional challenges of cancer, including emotion-focused coping, stress reduction, acceptance, and avoidance-based strategies.^12^, ^13^, ^14^ During active treatment, adolescents commonly draw on both cognitive and avoidant coping strategies, such as acceptance and cognitive reappraisal, to adapt to cancer-related stress.^15^^,^^16^ However, these studies have paid less attention to adolescents' role, involvement, and agency while engaging in these coping efforts. As individuals move beyond the acute phase of illness and transition into survivorship, they may reinterpret their cancer experiences and develop new understandings of their responses to illness over time.^17^^,^^18^ Therefore, examining survivors’ narrations and understandings of these earlier experiences may offer insight into dimensions of adolescent coping that contemporaneous accounts cannot fully capture.

Arthur Frank’s work on illness narratives provides a useful framework for understanding this process. Frank describes three broad forms of illness narratives — restitution, chaos, and quest — and suggests that individuals may make sense of illness by narrating and reorganizing their experiences over time.^19^ From this perspective, retrospective narratives may enable survivors not only to make sense of their experiences but also to articulate dimensions of agency, resistance, and personal involvement that may have been difficult to recognize or express during treatment itself.

To better understand retrospective interpretations of adolescent cancer experiences, the present study focused on young adult survivors who were at least two years post-treatment, a sufficient temporal distance for reflection and narrative reconstruction. Although some prior research has highlighted adolescents' preferences for participation, information, and involvement in their care,^20^ other studies have primarily focused on psychosocial, communication, and supportive care needs during treatment and survivorship.^21^, ^22^, ^23^ Comparatively less attention has been given to how young adult survivors retrospectively describe their own efforts to maintain agency, autonomy, and personal involvement while coping with cancer during adolescence. To address this gap, the present study retrospectively described young adult survivors' experiences of coping with cancer within the sociocultural context of Iran. An in-depth understanding of these retrospective narratives may contribute to more context-sensitive approaches that support adolescents’ agency, participation, and involvement during cancer treatment and survivorship.

## Methods

### Design

This qualitative study was conducted between February 2024 and July 2025 using conventional content analysis,^24^ an approach suited to exploring participants' experiences and perspectives without imposing predefined theoretical categories.^25^ This inductive approach enabled categories and interpretations to emerge directly from participants’ retrospective narratives of coping with cancer during adolescence.

### Participants and setting

Participants were initially recruited through purposive sampling, which was subsequently complemented by snowball sampling to access additional eligible participants. The study setting comprised tertiary hospitals affiliated with Birjand and Mashhad Universities of Medical Sciences in eastern Iran, where participants had previously received oncological care during adolescence.

The inclusion criteria were: (a) age 18–29 years at the time of interview, corresponding to the developmental period of emerging adulthood, which is characterized by identity exploration and significant life transitions,^26^ (b) having received a definitive cancer diagnosis during adolescence (11–18 years of age), (c) having completed treatment at least two years ago, to ensure sufficient temporal distance from the acute treatment period and to facilitate meaningful retrospective reflection on adolescent illness experiences, and (d) the willingness and ability to communicate and describe retrospective experiences related to adolescent cancer.

The exclusion criterion was severe psychiatric conditions that could interfere with participation in interviews or the capacity to provide coherent reflective accounts at the time of data collection. This was assessed through a combination of participant self-report, clinical history review, and medical record evaluation, conducted in consultation with pediatric oncologists involved in participants’ prior care.

Ultimately, 15 young adult cancer survivors retrospectively reflected on their adolescent cancer experiences (Table 1).

**Table 1:** Characteristics of young adult cancer survivors.

No.	Sex	Marital status	Type of cancer	Current age (years)	Age at disease onset (years)	Age at end of treatment (years)
P1	Male	Single	ALL	25	13	18
P2	Female	Single	ALL	21	12	13
P3	Female	Married	ALL	26	12	16
P4	Male	Single	Pancreatic adenocarcinoma	19	13	15
P5	Female	Married	Hodgkin lymphoma	25	12	14
P6	Male	Single	Hodgkin lymphoma	20	14	17
P7	Male	Single	Soft tissue sarcoma of the hand	21	11	13
P8	Male	Married	ALL	22	13	17
P9	Female	Single	ALL	20	16	18
P10	Male	Single	ALL	22	14	17
P11	Female	Single	ALL	20	11	13
P12	Male	Married	ALL	25	12	15
P13	Female	Married	ALL	20	13	16
P14	Male	Single	Hodgkin lymphoma	25	12	15
P15	Female	Married	ALL	23	12	14

ALL, Acute Lymphoblastic Leukemia.

### Data collection

Data were collected through in-depth, face-to-face interviews.^27^ Reflexivity was maintained throughout data collection through ongoing critical attention to the researchers’ assumptions, perspectives, and potential influence on both the interview process and the emerging analysis. Data collection began with open-ended, unstructured interviews, which were designed to encourage participants to share narratives of their adolescent experiences with cancer. As data analysis and collection proceeded concurrently and initial concepts began to emerge, semi-structured interviews gradually evolved. This flexible approach facilitated detailed descriptions of experiences related to the period of active treatment while allowing emerging concepts to be explored in greater depth.

Although an interview guide was developed to ensure systematic exploration of key topics, the ordering and phrasing of questions were flexibly adjusted in response to the flow and content of each interview. Interviews commenced with the following open-ended question:

- Would you please describe your experiences and challenges during adolescent cancer and how you coped with them?

Probing and follow-up questions were subsequently employed to clarify meanings, explore nuances, and encourage richer descriptions of participants' experiences. Interviews were conducted at locations chosen by participants to promote comfort and openness, and lasted between approximately 45–90 minutes. All interviews were audio-recorded with participants’ informed consent. The interviewer recorded field notes to capture nonverbal cues and contextual observations about the interview setting immediately after each interview.

Data collection and analysis were conducted concurrently. Preliminary saturation was identified after the thirteenth interview, when no new categories emerged from the data.^27^^,^^28^ Two additional interviews were conducted to confirm saturation. Decisions regarding saturation were made through ongoing discussion among the research team throughout the analytic process.

### Data analysis

Data analysis and collection proceeded concurrently, following the conventional content analysis approach described by Graneheim and Lundman.^24^ Each interview was transcribed verbatim shortly after completion. Audio recordings were listened to multiple times, and transcripts were read several times to develop an overall sense of participants’ retrospective narratives. Transcripts were imported into MAXQDA 2022 to organize and manage the data.

The interview texts were then divided into meaning units—words, sentences, or paragraphs —that conveyed content relevant to the study’s aim. Each meaning unit was condensed while preserving its essential meaning. The condensed meaning units were then abstracted and assigned initial codes that reflected their content.

To strengthen analytical rigor, the first author and a second researcher— both experienced in qualitative research — independently reviewed and coded approximately 20% to 25% of the initial interview transcripts. The independently generated codes were then compared and discussed in research team meetings, and any discrepancies in coding or interpretation were resolved through reflective dialogue and consensus. This process informed the collaborative development and refinement of a preliminary coding framework, comprising initial codes and their operational definitions.

The first author subsequently analyzed the remaining interviews using this evolving coding framework. Throughout the analysis process, data were continuously compared with existing codes, and new codes were added or refined when necessary. Qualitative research experts and research team members regularly discussed how to organize codes into subcategories and broader categories based on their similarities and differences.

### Trustworthiness

To ensure the trustworthiness of the findings, this study adhered to Lincoln and Guba’s criteria,^29^ including credibility, dependability, confirmability, and transferability.

Credibility was supported through several strategies. Prolonged engagement with the data was achieved by repeatedly listening to audio recordings and reviewing interview transcripts across multiple analytical cycles. Reflexivity was maintained throughout the research process via ongoing critical examination of the researchers’ assumptions and their potential influence on data collection, interpretation, and analysis. Peer debriefing, facilitated by regular research team discussions and periodic consultation with experienced qualitative researchers, further strengthened the development and refinement of codes, subcategories, and categories. Member checking was conducted with 5 participants, who reviewed selected summaries and illustrative excerpts. Their feedback confirmed that the interpretations reflected their experiences, and no major changes were required.

Dependability was ensured through systematic documentation of all research stages, including analytical decisions and coding procedures, to guarantee transparency and consistency. Collaborative discussions regarding coding and interpretation among research team members and qualitative researchers further strengthened the dependability of the analytical process. Discrepancies in interpretation were addressed through reflective dialogue and iterative team discussion. Confirmability was established by maintaining a comprehensive audit trail that documented analytical decisions, field notes, reflective memos, and the progression from raw data to final categories. This documentation allowed the analytical process to be traced and evaluated independently. Transferability was supported by providing rich, detailed descriptions of the research context, study setting, participant characteristics, and analytical procedures. Purposive sampling with maximum variation enabled the inclusion of participants with diverse cancer diagnoses, treatment histories, and demographic backgrounds, thereby allowing readers to judge the applicability of the findings to comparable contexts.

## Results

Fifteen young adult cancer survivors participated in this study (Table 1). Analysis of their experiences yielded four main categories and 14 subcategories (Table 2). The findings illustrated how participants retrospectively responded to the psychological, social, and physical challenges of cancer to sustain hope, preserve personal control, manage social relationships, and remain actively involved in treatment and everyday life. These narratives depicted an active, self-directed process of adolescent coping aimed at navigating illness while sustaining continuity in everyday functioning and social connection.

**Table 2:** Categories and subcategories.

Categories	Subcategories
Sustaining hope despite suffering	Reinventing hope
	Asserting adolescent authority
	Using humor and distraction
	Enduring difficult treatment with pride
Maintaining control under illness constraints	Seeking illness information
	Resisting treatment-related changes
	Maintaining independence
Managing social relationships	Managing demoralizing reactions
	Normalizing appearance changes
	Drawing strength from emotional support
	Rebuilding relationships
Managing life responsibilities	Taking treatment responsibility
	Protecting health
	Maintaining daily functioning

### Sustaining hope despite suffering

Young adult survivors described sustaining hope as an active and ongoing endeavor amid significant physical and emotional suffering. This category comprised four subcategories: Reinventing Hope, Asserting Adolescent Authority, Using Humor and Distraction, and Enduring Difficult Treatment with Pride. Participants described feelings of personal capability, determination, and inner strength to maintain hope throughout their illness. Sustaining hope was actively constructed and maintained in the face of difficult treatment experiences.

#### Reinventing hope

Participants described consciously working to make their illness more bearable by reframing its significance in their daily lives and transforming suffering into an experience that could carry personal meaning. In their retrospective accounts, participants actively constructed and sustained hope to maintain morale and resist negative thinking during treatment.

“I never thought about my disease until my recovery; I just kept telling myself that it is a small lump, not cancer.” (Participant 1)

“I once heard about a track and field champion who became a world champion with a sprained ankle, so maybe there is something positive in it for me.” (Participant 4)

“At that time, everything had a different meaning for me like the people around me, birds, the wind, or even the windows of my room, and I took many photos of these scenes.” (Participant 8)

#### Asserting adolescent authority

Survivors recalled assuming responsibilities beyond their chronological age during treatment and approached illness with a strong sense of determination and personal authority.

“Even though I was 15, I was behaving like a 20-year-old person; my childhood had completely disappeared.” (Participant 2)

Several participants also described making deliberate efforts to remain psychologically strong during treatment:

“I kept telling myself that I had to stay strong and get through this period without losing hope.” (Participant 7)

#### Using humor and distraction

Participants frequently recalled using humor and everyday distractions as strategies to cope with the distress and monotony of treatment. These strategies helped them shift their attention away from pain, fear, and the clinical environment.

“Sometimes I tried to keep myself busy with different things so that I wouldn’t constantly think about the disease.” (Participant 6)

“I used to make jokes about my situation in front of my family and friends to change the atmosphere.” (Participant 5)

#### Enduring difficult treatment with pride

Participants described enduring prolonged and demanding treatment with patience and a sense of pride. They recalled interpreting patience not merely as a passive acceptance, but as a marker of personal maturity and strength. This framing of endurance appeared particularly salient within the Iranian sociocultural context, in which strength, stoicism, and resilience carry significant cultural values.

“Although I waited for the intrathecal chemotherapy procedure outside the operating room from 9 a.m. to 12:30 p.m., it was unsuccessful. However, I endured it because I did not want my mother to become worried about me anymore.” (Participant 15)

Some male participants were under pressure to hide pain, which may reflect sociocultural expectations surrounding masculinity and emotional expression:

“During IV insertion, when I cried, nurses would tell me ‘you are a man now and you shouldn’t cry.’ Since I was older than the other children there, I felt ashamed of crying and kept telling myself: this is embarrassing—you’re a man, you shouldn’t cry.” (Participant 6)

Participants also recalled actively resisting the constraints that illness imposed on their sense of control and autonomy.

#### Maintaining control under illness constraints

This category highlighted their efforts to maintain personal control in the face of illness-related limitations. These accounts reflected persistent attempts to preserve a sense of autonomy and agency over their bodies, treatment decisions, and daily life. Participants described these experiences through three interrelated subcategories: Seeking Illness Information, Resisting Treatment-Related Changes, and Maintaining Independence.

#### Seeking illness information

Participants described actively seeking information about their illness, particularly when they perceived that information was being withheld. They attempted to better understand their condition by searching for medical information and interpreting available clues.

“The diagnosis was written on the board above my bed. I searched it on the internet and found that the disease could be either malignant or benign, and that chemotherapy was necessary.” (Participant 13)

“I knew my illness was serious, so whenever the nurses left the room, I would pick up my medical file and read what had been written.” (Participant 8)

#### Resisting treatment-related changes

Participants also recalled actively resisting the physical changes imposed by treatment, which they described as significant threats to their sense of identity and personal integrity. Hair loss, in particular, was frequently cited as a deeply distressing experience that many participants were unwilling to accept without resistance.

“I was told to shave my head because of my hair loss, but I refused it and lost my hair naturally. I felt like a part of me was being torn away.” (Participant 2)

“I was very stubborn and did not listen to anyone. I did not want to accept the treatment and drugs when I was hospitalized for chemotherapy for the first time; however, they could convince me.” (Participant 4)

#### Maintaining independence

Participants also described trying to maintain a sense of competence by minimizing reliance on others. This pursuit of autonomy occasionally included declining professional psychological support.

“Although there were psychologists in the hospital to advise us, I was confident enough about myself that I needed no therapist.” (Participant 9)

“I created a strong character in my mind that always guided me, so I needed no one else.” (Participant 7)

These experiences were often accompanied by efforts to preserve social relationships and participation despite illness-related challenges.

### Managing social relationships

Social relationships during illness were characterized by ongoing challenge and active navigation. Participants described persistent efforts to remain connected to their social world and preserve their sense of belonging, despite the social disruptions caused by visible physical changes, altered reactions from others, and concerns about stigma. These efforts were reflected in four interrelated subcategories: Managing Demoralizing Reactions, Normalizing Appearance Changes, Drawing Strength from Emotional Support, and Rebuilding Relationships.

#### Managing demoralizing reactions

This subcategory captured adolescents' conscious and deliberate efforts to protect their psychological well-being from discouraging and stigmatizing social reactions. Participants described employing several active social management strategies: selectively limiting interactions with peers who lacked understanding of their illness, making deliberate choices about how to respond to unwanted attention, and cultivating a stance of adaptive indifference to buffer the emotional impact of others’ reactions.

“I really hated it when some people looked at me pitifully; however, I stayed silent and said nothing because I did not want to hurt anyone’s feelings.” (Participant 4)

“One of my friends described me as an ugly person, but I just laughed and was indifferent.” (Participant 7)

#### Normalizing appearance changes

Participants described being highly sensitive to others’ gaze and attempting to re-establish a sense of normality despite visible signs of illness. Fear of stigma and pity was frequently remembered as a major reason for concealing illness-related changes.

“I did not want other children to know about my illness because they might change their behaviors toward me.” (Participant 6)

“I wore a fur coat so people would think I had hair.” (Participant 10)

Over time, participants described a gradual process through which physical changes became more normalized and hypersensitivity to others’ reactions diminished. Notably, peer solidarity within the hospital ward — being among others facing similar experiences — emerged as a meaningful context for this process of normalization and acceptance.

“In the ward, no one was superior to anyone else because everyone was bald.” (Participant 2)

#### Drawing strength from emotional support

Young cancer survivors described emotional support from others as an important source of psychological strength throughout their illness experience. Family solidarity was remembered as a meaningful expression of support.

“I realized my uncles' support when they shaved their heads at the same time as I did.” (Participant 8)

“I wanted people around me to treat me as they always had and to believe that I would get better.” (Participant 5)

Empathetic support from friends during recurrence was also remembered as emotionally meaningful.

“A glimmer of hope appeared when some of my friends had shaved their heads. I told myself: I got through it once before; I can do it again.” (Participant 14)

#### Rebuilding relationships

The experience of illness gradually moved participants toward rebuilding and strengthening their social relationships. Participants described maintaining contact with peers and school staff despite prolonged absence from everyday life.

“During my illness, I stayed in contact with my school principal and even with friends I had not seen for a long time.” (Participant 15)

“Since I was a bit older than the other children, I would go and comfort those who were afraid of IV insertion.” (Participant 1)

“At first, I didn’t interact much with the nurses, but later I learned how to communicate with them; I asked their names and joked with them.” (Participant 9)

### Managing life responsibilities

The final category captured participants’ retrospective accounts about their active and responsible roles in managing both the demands of treatment and everyday life. This category encompassed three interrelated subcategories: Taking Treatment Responsibility, Protecting Health, and Maintaining Daily Functioning.

#### Taking treatment responsibility

Participants described responsibility for treatment-related demands and maintaining active involvement in the treatment process. Treatment was often viewed as a necessary pathway toward recovery.

“While I traveled with my mother to Mashhad for chemotherapy, I felt that the entire burden of this journey was on my shoulders because she was illiterate.” (Participant 5)

“I used to tell myself that I should go through it in a way that does not make it too difficult for me.” (Participant 7)

#### Protecting health

Participants described extending their sense of responsibility into everyday life through efforts to protect their health during illness. These accounts reflected deliberate decision-making regarding self-protection and avoidance of situations perceived as threatening to their health.

“Although I trust in God, I should not go where there is infection or viral transmission.” (Participant 14)

#### Maintaining daily functioning

Participants also described attempting to continue their everyday life despite treatment-related disruptions. Educational activities and daily functioning were particularly emphasized in their accounts.

“Although I had not gone to school for about a year because of my illness, I self-studied my lessons to reach my friends' levels of achievement.” (Participant 11)

“I had no one to guide me, but I repeated the materials several times until I learned them.” (Participant 11)

These categories were not experienced or retrospectively described as isolated or disconnected aspects of illness. Rather, they represented interconnected dimensions of an overarching pattern: participants' active, purposeful efforts to maintain agency, preserve continuity, and sustain meaningful engagement in everyday life and social relationships in the face of cancer. Across all four categories, survivors consistently portrayed themselves as active agents who drew on inner resources, social connections, and personal responsibility to navigate cancer during a critical developmental period.

## Discussion

This study retrospectively described young adult cancer survivors’ experiences of coping with cancer during adolescence. Narratives portrayed coping as an active, ongoing process to sustain hope, preserve a sense of control, maintain social connections, and remain engaged in everyday responsibilities despite illness-related challenges. Across all four categories, participants actively responded to the psychological, social, and practical challenges associated with cancer during a critical developmental period.

The retrospective nature of the interviews added a further dimension to these accounts. With time elapsed since treatment, participants were able to reflect on how they had navigated illness-related challenges and later made sense of their adolescent cancer experiences. These narratives, therefore, offered insight not only into coping during treatment, but also into how survivors reconstructed and interpreted those experiences over time.

The first category, sustaining hope despite suffering, reflects how participants retrospectively maintained their hope through ongoing psychological effort during their illness. Participants tried to make suffering more bearable through self-reassurance, positive reinterpretation of difficult experiences, and deliberate attention to meaningful aspects of everyday life during treatment. These accounts suggest that sustaining hope during adolescent cancer involved continuous effort to remain emotionally engaged and future-oriented despite ongoing suffering. According to Snyder’s hope theory, hope consists of agency thinking — the motivational drive to pursue desired goals — and pathways thinking — the perceived ability to find routes toward those goals.^30^

Participants’ efforts to reinterpret illness as manageable and maintain focus on future recovery appear consistent with both dimensions of this framework. Prior qualitative research with adolescents and young adults with cancer has similarly described hope as an active psychological resource that patients work to sustain throughout treatment.^31^

Participants also retrospectively approached illness with a strong sense of determination and personal resolve. Several participants described responsibilities that felt beyond their chronological age, framing the challenge of illness as something requiring active engagement rather than passive endurance. In their accounts, maintaining psychological determination appeared closely connected to hope, some participants explicitly told themselves to remain strong as a means of getting through treatment without losing hope. Similar patterns of self-directed determination and personal agency during cancer treatment have been documented in qualitative research with adolescents and young adults during active treatment, in which participants described developing a mental mindset oriented toward actively confronting their cancer and advocating for themselves in day-to-day decisions.^32^

Participants frequently recalled using humor and distraction as deliberate strategies to cope with the distress and monotony of treatment. These approaches helped participants shift their attention away from pain, fear, and the clinical environment. Humor, in particular, appeared to help them manage individual distress and change the emotional atmosphere among family members and friends during difficult periods of treatment. Qualitative research with adolescents and young adults facing cancer has similarly identified distraction and efforts to focus on the present as coping strategies used during treatment.^33^

Participants also described enduring difficult treatment as a source of pride, maturity, and psychological strength. Several participants framed patience and endurance during treatment as evidence of personal capability. Maintaining composure during painful procedures helped certain participants preserve a sense of dignity and inner strength. Some male participants described feeling pressure to appear strong and avoid showing pain within family and clinical environments, which may reflect broader gendered expectations regarding emotional expression.^34^ Emotional endurance functioned not only as a means of coping with treatment-related challenges, but also as a meaningful way of maintaining self-respect, determination, and hope throughout prolonged illness.

The second category, maintaining control under illness constraints, reflects participants' sense of personal involvement and autonomy despite illness-related limitations. Self-determination theory identifies autonomy and competence as important psychological needs.^35^ Participants’ accounts in this category suggested that treatment-related restrictions often challenged their sense of independence, and that many of the behaviors they described reflected their efforts to remain actively involved in their own care and daily decision-making.

One aspect of this pattern involved participants’ active pursuit of information about their condition. Participants described searching online for information and reviewing their medical records, particularly when they perceived that information was being withheld by health care providers or family members. Access to information was important for participating in their own care during a period marked by uncertainty and limited control. Previous qualitative research has similarly documented that adolescents with cancer value honest information and may experience distress — including fear and loneliness — when truthful communication feels restricted.^36^ Notably, while participants preferred receiving information from human sources over the internet, participants in the present study described actively seeking information online when they perceived that health care providers or family members were withholding information. This pattern may reflect differences in information accessibility or the perceived trustworthiness of available sources within their treatment context.

Participants also described resisting certain illness-related changes, most notably refusing to shave their heads following hair loss and initially resisting aspects of treatment routines. As adolescence is closely associated with identity development and continuity of self,^37^ visible treatment-related changes may hold particular emotional significance during this stage. Most of the participants ultimately accepted treatment, suggesting that their resistance was directed more toward illness-related disruptions than toward treatment itself. A qualitative study of adolescents and young adults with cancer confirmed similar efforts to navigate cancer in a self-directed manner.^38^ The present findings suggest that resisting illness-related disruptions may have been one way of preserving personal continuity and a recognizable sense of self during treatment.

Participants also described deliberate efforts to manage illness-related challenges independently and minimize reliance on professional psychological support available within the hospital. These accounts appeared less reflective of rejecting support altogether and more related to maintaining a sense of competence and personal involvement during illness. Some participants described relying on inner strength and self-management to cope with emotionally difficult situations while limiting dependence on others where possible. While qualitative research has documented the importance of independence among adolescent and young adult cancer survivors, particularly in the context of balancing health management and everyday life following treatment,^39^ the present findings suggest that maintaining independence may also be important during active treatment. In the present study, participants described self-reliance as a way of preserving a sense of personal competence and agency while coping with the immediate demands and dependencies associated with treatment.

The third category, managing social relationships, reflects how participants retrospectively described navigating the social dimensions of cancer through ongoing interpersonal adjustment and deliberate social management. Rather than withdrawing completely from social life, participants described selectively managing interactions, seeking emotional support, and gradually rebuilding relationships throughout their illness experience. These accounts suggest that social challenges during adolescent cancer were experiences requiring active negotiation to preserve social connection and personal involvement during treatment.

Participants described managing challenging social reactions through selective social engagement. Some limited interactions with peers who were uncomfortable with or lacked understanding of their illness, while others responded to pitying looks or hurtful comments with silence, humor, or deliberate indifference. These responses helped reduce emotional distress and maintain some degree of social participation during treatment. Participants regulated social interactions in ways that helped protect their emotional well-being while preserving connection with their social environment. Although previous qualitative research has identified peer relationships as a treatment-related barrier to connection,^40^ the present findings suggest a more active approach to managing social relationships; participants described deliberately regulating their social exposure as a way of protecting their emotional well-being while remaining connected to their social world.

Participants also described deliberate efforts to manage visible illness-related changes, particularly hair loss, by minimizing signs of illness in social settings. Concerns about how others might perceive these changes appeared to shape many of these responses. Several participants worried that visible bodily changes would alter how others treated them, leading some to conceal appearance-related changes or avoid drawing attention to their illness. While previous qualitative research has explored how AYA cancer survivors retrospectively reflect on the impact of treatment on their physical appearance and sense of social normalcy,^41^ the present findings suggest that concerns about visible bodily changes shaped participants’ social behaviors during active treatment. Appearance management appeared to function as a deliberate strategy for navigating social environments during illness.

Importantly, adaptation to appearance-related changes was not described solely as an individual process. Being surrounded by peers with similar treatment-related changes within the hospital ward could reduce feelings of difference and support gradual acceptance of altered appearance. Shared experiences within the treatment environment may have fostered a sense of belonging, making appearance-related differences less disruptive to participants’ social adjustment.

Emotional support from family members, peers, and health care providers emerged as an important source of strength throughout participants' illness experiences. Participants particularly valued forms of support that conveyed acceptance, confidence, and ordinary relational connection rather than pity or excessive concern. Gestures of solidarity, such as family members shaving their heads in support, were experienced as especially meaningful because they reflected shared involvement rather than distant sympathy. These findings suggest that the perceived value of support depended not only on its availability but also on how it was communicated. Previous qualitative research has similarly emphasized the importance of emotional support and meaningful peer relationships during cancer treatment.^23^^,^^40^ The present findings extend this literature by suggesting that support was experienced as particularly meaningful when it reinforced participants’ sense of acceptance, belonging, and continued social connection.

Finally, participants described how illness gradually contributed to rebuilding and strengthening social relationships over time. Maintaining contact with friends and school staff, developing closer communication with health care providers, and supporting younger patients reflected increasing interpersonal engagement over time. For some participants, supporting other patients provided not only emotional meaning but also a renewed sense of usefulness and social value when ordinary social roles had been disrupted. While previous studies have primarily examined adolescents and young adults as recipients of support during cancer treatment,^23^^,^^40^ the present findings additionally suggest that participants played an active role in shaping supportive relationships within hospitals.

The fourth and final category, managing life responsibilities, reflects how participants retrospectively described actively handling both treatment demands and everyday responsibilities during illness. Participants described treatment-related responsibilities as part of their own effort to manage illness and preserve continuity in daily life, often approaching treatment pragmatically and purposefully rather than as an externally imposed obligation. They recalled attempting to remain active contributors to their own care and daily functioning despite the limitations imposed by illness.

Participants also recalled consciously avoiding situations they perceived as health threats, such as crowded environments or settings associated with infection risk. These accounts suggest that participants were trying to protect their health while managing the uncertainty and dependency associated with serious illness. Previous studies from comparable contexts have primarily examined survivors' perceptions of ongoing health vulnerability after treatment completion.^42^ In contrast, the present findings suggest that concerns about physical vulnerability may also influence how adolescents actively manage health-related risks during treatment.

Another dimension of participants' experiences involved efforts to preserve continuity in everyday life despite treatment-related disruptions, particularly through maintaining schoolwork and ordinary routines during prolonged absences from school. These activities appeared to help participants maintain connection with ordinary adolescent life and preserve a sense of continuity during illness. Participants often described developing these strategies independently and attempting to adapt daily life around treatment demands as much as possible. Qualitative research with teenagers with cancer has similarly found that engagement with educational activities during illness carries important developmental and social meaning for young people navigating treatment.^43^

### Implications for nursing practice and research

The findings of this study have several implications for nursing and clinical practice. The findings suggest that adolescents with cancer may benefit when nursing professionals recognize them as active participants in their own care rather than passive recipients of treatment. Nurses and health care providers should create opportunities for developmentally appropriate communication, providing honest, age-appropriate information and actively involving adolescents in treatment-related discussions and decisions. Such approaches may help strengthen trust and support adolescents' sense of autonomy and participation during treatment. Nurses should also recognize and respect adolescents' efforts to manage aspects of their care independently, rather than interpreting such behaviors solely as resistance or noncompliance.

Health care providers should also attend to the social dimensions of the adolescent cancer experience. Peer relationships, appearance-related concerns, and changes in body image may affect adolescents' self-confidence and social participation during treatment. Creating space for adolescents to discuss these concerns and, where possible, facilitating peer connections can help preserve their sense of belonging and social continuity. Regarding family involvement, nurses should support families in balancing emotional care with respect for adolescents' need for involvement and self-direction in their own care.

Where clinically feasible, nurses and multidisciplinary teams should support adolescents in maintaining school attendance, educational engagement, and ordinary daily routines. Helping adolescents stay connected to schoolwork, peer relationships, and everyday activities during treatment may help them maintain a sense of continuity with normal adolescent life throughout the cancer experience.

The findings also have implications for future research. Given the retrospective nature of the present study, longitudinal research involving adolescents during active treatment may help clarify how coping strategies, autonomy, social relationships, and everyday participation evolve over time. Future studies across diverse cultural settings may help explore how sociocultural contexts shape adolescents' experiences of coping with cancer and inform the development of culturally responsive supportive care interventions.

### Limitations

This study has several limitations that should be considered when interpreting the findings. First, the findings were derived from retrospective accounts of young adult survivors reflecting on their adolescent experiences. Later life experiences, personal development, and the reinterpretation of earlier experiences may have influenced these narratives. Second, the study included only survivors who had completed treatment and were willing to participate in interviews. Consequently, the findings primarily reflect the perspectives of survivors who successfully transitioned into young adulthood, potentially overlooking adolescents with different illness trajectories, severe late effects, or those who did not survive cancer.

In addition, participants were recruited from tertiary hospitals in eastern Iran, and the findings are therefore situated within a specific sociocultural and health care context. Although this contextual focus provides important insight into culturally situated experiences of adolescent cancer, it may limit the transferability of the findings to other sociocultural and health care settings. Finally, the study was based solely on survivors' retrospective perspectives and excluded the views of parents, health care providers, or other family members. Future qualitative studies using longitudinal and multi-perspective approaches could provide a more comprehensive understanding of adolescents' cancer experiences across both treatment and survivorship phases.

## Conclusions

This study explored how young adult cancer survivors retrospectively described their experiences of coping with cancer during adolescence. Participants attempted to preserve hope, autonomy, social connectedness, and involvement in everyday life throughout their illness experience. Rather than portraying themselves as passive recipients of care, they described actively engaging with the physical, emotional, and social challenges of cancer while attempting to maintain a sense of personal agency during treatment.

The findings underscore the importance of recognizing adolescents with cancer not only as patients receiving treatment but also as individuals actively working to preserve their relationships, participation in everyday life, and sense of personal agency. Supporting adolescents’ involvement in their care, providing honest, developmentally appropriate communication, and attending to their social and developmental needs may contribute to more person-centered approaches to adolescent cancer care.

The findings also contribute to the growing qualitative literature on adolescent cancer by offering retrospective narrative accounts that illuminate not only the challenges adolescents experienced during cancer but also how they retrospectively made sense of and navigated those experiences. By presenting these accounts from young adult survivors in Iran, this study contributes to a broader and more geographically and culturally diverse qualitative evidence base on adolescent cancer experiences.

## CRediT authorship contribution statement

Marzieh Helalbirjandi: Conceptualization, Methodology, Investigation, Data Curation, Formal Analysis, Writing – Original Draft. Ahmad Nasiri: Conceptualization, Methodology, Supervision, Project Administration, Writing – Review & Editing. Gholamhossein Mahmoudirad: Validation, Formal Analysis, Writing – Review & Editing. Ali Ghasemi: Resources, Validation, Investigation, Writing – Review & Editing. All authors have read and approved the final manuscript and agreed to be accountable for all aspects of the work.

## Ethics statement

This study was approved by the Ethics Committee of Birjand University of Medical Sciences (Approval No. IR.BUMS.REC.1403.104) and was conducted in accordance with the 1964 Declaration of Helsinki and its later amendments, or comparable ethical standards. All participants provided written informed consent and agreed to audio recording before participation. Participants were informed that their data would remain confidential and that they could stop the interview or withdraw from the study at any time without any negative consequences.

## Data availability statement

The data that support the findings of this study are available on request from the corresponding author, A.N. The data are not publicly available because they contain information that could compromise the privacy of research participants.

## Declaration of generative AI and AI-assisted technologies in the writing process

During the preparation of this manuscript, the authors used ChatGPT and Grammarly for English language editing only. After using these tools, the authors reviewed and edited the content and take full responsibility for the accuracy and integrity of the final manuscript.

## Funding

This study was supported by the Vice Chancellor for Research and Technology of Birjand University of Medical Sciences (Grant No. 457437). The funders had no role in the study design or in the collection, analysis, interpretation of data, writing of the report, or decision to submit the article for publication.

## Declaration of competing interest

The authors declare no conflict of interest.
